# Glucose Tightly Controls Morphological and Functional Properties of Astrocytes

**DOI:** 10.3389/fnagi.2016.00082

**Published:** 2016-04-18

**Authors:** Chun-Yao Lee, Glenn Dallérac, Pascal Ezan, Miroslava Anderova, Nathalie Rouach

**Affiliations:** ^1^Neuroglial Interactions in Cerebral Physiopathology, Center for Interdisciplinary Research in Biology, Collège de France, Centre National de la Recherche Scientifique UMR 7241, Institut National de la Santé et de la Recherche Médicale U1050, Labex Memolife, PSL Research UniversityParis, France; ^2^Department of Cellular Neurophysiology, Institute of Experimental Medicine, Academy of Sciences of the Czech RepublicPrague, Czech Republic; ^3^Department of Neuroscience, 2nd Faculty of Medicine, Charles UniversityPrague, Czech Republic

**Keywords:** hippocampus, astrocytes, neuroglial interactions, glucose, energy deprivation, connexins, volume, calcium

## Abstract

The main energy source powering the brain is glucose. Strong energy needs of our nervous system are fulfilled by conveying this essential metabolite through blood *via* an extensive vascular network. Glucose then reaches brain tissues by cell uptake, diffusion and metabolization, processes primarily undertaken by astrocytes. Deprivation of glucose can however occur in various circumstances. In particular, ageing is associated with cognitive disturbances that are partly attributable to metabolic deficiency leading to brain glycopenia. Despite the crucial role of glucose and its metabolites in sustaining neuronal activity, little is known about its moment-to-moment contribution to astroglial physiology. We thus here investigated the early structural and functional alterations induced in astrocytes by a transient metabolic challenge consisting in glucose deprivation. Electrophysiological recordings of hippocampal astroglial cells of the *stratum radiatum* *in situ* revealed that shortage of glucose specifically increases astrocyte membrane capacitance, whilst it has no impact on other passive membrane properties. Consistent with this change, morphometric analysis unraveled a prompt increase in astrocyte volume upon glucose deprivation. Furthermore, characteristic functional properties of astrocytes are also affected by transient glucose deficiency. We indeed found that glucoprivation decreases their gap junction-mediated coupling, while it progressively and reversibly increases their intracellular calcium levels during the slow depression of synaptic transmission occurring simultaneously, as assessed by dual electrophysiological and calcium imaging recordings. Together, these data indicate that astrocytes rapidly respond to metabolic dysfunctions, and are therefore central to the neuroglial dialog at play in brain adaptation to glycopenia.

## Introduction

Glucose is the main metabolic fuel of the brain. Cerebral activities indeed consume high amounts of glucose, reaching nearly 20% of total body consumption (Shulman et al., 2004; Lord et al., 2013; Mergenthaler et al., 2013). To meet this strong energy demand, glucose is provided by the blood *via* a widespread vascular network and is distributed in brain tissue through cell uptake, diffusion and metabolization (Harris et al., 2012; Magistretti and Allaman, 2015).

Remarkably, astrocytes are long known to play a crucial role in all these processes, thanks to abundant expression in their perivascular domains of glucose transporters, gap junction channels and enzymes metabolizing glucose (Rouach et al., 2008; Allaman et al., 2011; Escartin and Rouach, 2013; Magistretti and Allaman, 2015). Thereby, astrocytes largely contribute to on-demand energy supply of active neurons by providing a physical link *via* their perivascular endfeet between the blood vessels and synaptic terminals (Tsacopoulos and Magistretti, 1996; Kacem et al., 1998; Simard et al., 2003). In particular, it has extensively been reported that neurons obtain part of their energy from extracellular lactate, a glucose metabolite produced by astrocytes (Tsacopoulos and Magistretti, 1996; Schurr et al., 1999; Rouach et al., 2008). Lactate is indeed required for synaptic activity (Rouach et al., 2008), long-term memory (Suzuki et al., 2011; Dallérac and Rouach, 2016) or conditioned responses to cocaine (Boury-Jamot et al., 2015), although some studies reported that neurons can also directly uptake and use glucose (Itoh et al., 2004; Nehlig et al., 2004; Barros et al., 2009; Jakoby et al., 2014; Patel et al., 2014; Lundgaard et al., 2015).

Despite high glucose consumption, brain access to glucose is limited since the latter cannot be synthetized locally or stored in significant amount in the form of glycogen (Wender et al., 2000; Brown, 2004; Brown et al., 2005; Mergenthaler et al., 2013). The brain is therefore particularly sensitive to shortage of blood glucose supply, which can rapidly alter information processing by neuroglial networks (Papadopoulos et al., 1997; Ioudina et al., 2004; Harris et al., 2012; Howarth et al., 2012). Deprivation of glucose can occur in various circumstances, such as during sustained neuronal activity, when glucose demand exceeds supply, during fasting or in patients with ischemia or diabetes with intensive glycemia control. Importantly, ageing is associated with cognitive disturbances that are partly attributable to brain metabolic deficiency (Korol and Gold, 1998; Abdelhafiz et al., 2015). In particular, recurrent glycopenia due to a strong reduction in glucose diffusion through the extracellular fluid has been found to impair memory formation (McNay and Gold, 2001; Sykova, 2001; Sykova et al., 2002; McNay, 2005).

Although it is admitted that glucose and its metabolites are crucial to sustain neuronal activity (Tsacopoulos and Magistretti, 1996; Schurr et al., 1999; Ioudina et al., 2004; Rouach et al., 2008; Allaman et al., 2011; Suzuki et al., 2011; Harris et al., 2012; Mergenthaler et al., 2013; Boury-Jamot et al., 2015; Lundgaard et al., 2015), much less is known about the acute role of glucose in astroglial physiology. We thus here investigated the early structural and functional alterations induced in astrocytes by a transient metabolic challenge consisting in glucose deprivation. We found that glucose deprivation specifically increases astrocyte membrane capacitance, while it has no impact on other passive membrane properties of hippocampal astrocytes *in situ*. Consistent with this change, glucose deprivation relatively rapidly increases astrocyte cell volume. Furthermore, we demonstrate that acute glucose deficiency also alters typical functional properties of astrocytes. We indeed found that glucose deprivation decreases their gap junction-mediated intercellular communication, known to regulate astroglial volume (Pannasch et al., 2011; Chever et al., 2014), while it increases their intracellular calcium levels in correlation with downregulation of synaptic activity. These data indicate that astrocytes are integral and early targets of metabolic dysfunctions, which are therefore expected to rapidly compromise the neuroglial dialog at play for efficient processing of brain information.

## Materials and Methods

### Animals

Experiments were carried out according to the guidelines of European Community Council Directives of 01/01/2013 (2010/63/EU) and our local animal care committees (Center for Interdisciplinary Research in Biology in College de France (France) and Institute of Experimental Medicine (IEM; Czech Republic). Experiments were performed on the hippocampus of wildtype (C57BL6/J) mice and GFAP-eGFP mice, in which the enhanced green fluorescent protein (eGFP) is expressed under the control of the promoter for human glial fibrillary acidic protein (GFAP; Nolte et al., 2001). All efforts were made to minimize the number of used animals and their suffering. For all analyses, mice of both genders and littermates were used at 3–4 weeks of age.

### Acute Brain Slices

Acute transverse brain and hippocampal slices (400 μm) were prepared as previously described (Benesova et al., 2012; Pannasch et al., 2014). Slices were maintained at room temperature in a storage chamber containing a standard artificial cerebrospinal fluid (ACSF (in mM): 119 NaCl, 2.5 KCl, 2.5 CaCl_2_, 1.3 MgSO_4_, 1 NaH_2_PO_4_, 26.2 NaHCO_3_, and 11 glucose, saturated with 95% O_2_ and 5% CO_2_) for at least 1 h before the experiments. For the glucose-deprivation experiments, glucose (11 mM) was substituted by sucrose (11 mM) to maintain ACSF osmolarity.

### Electrophysiology

Slices were transferred in a submerged recording chamber mounted on an Olympus BX51WI microscope equipped for infrared-differential interference (IR-DIC) microscopy and were perfused with standard ACSF (2 ml/min). All experiments were performed in CA1 *stratum radiatum*. Field excitatory postsynaptic potentials (fEPSPs) and whole-cell patch-clamp recordings of astrocytes were performed in the CA1 *stratum radiatum* region of the hippocampus. fEPSPs were recorded with glass pipettes (2–5 MΩ) filled with ACSF. Postsynaptic responses were evoked by stimulating Schaffer collaterals (0.1 Hz) in CA1 *stratum radiatum* with ACSF filled glass pipettes. Whole-cell recordings were obtained from *stratum radiatum* astrocytes using 4–6 MΩ glass pipettes filled with (in mM): 105 K-gluconate, 30 KCl, 10 HEPES, 10 phosphocreatine, 4 ATP-Mg, 0.3 GTP-Tris, 0.3 EGTA (pH 7.4, 280 mOsm). Astrocytes were identified by their small somata, low input resistance and resting membrane potential, passive membrane properties (linear IV relationship), lack of action potential and extensive gap junctional coupling. For intercellular dye coupling experiments, the internal solution contained biocytin (7 mg/ml), which diffused passively in astrocytes patched in current-clamp mode during 30 min. Recordings were acquired with MultiClamp 700B amplifier (Molecular Devices), digitized at 10 kHz, filtered at 2 kHz, stored and analyzed on computer using Clampex10.3 and Clampfit10.3 Softwares (Molecular Devices). Biocytin was obtained from Sigma, and all other chemicals were from Tocris.

### Immunohistochemistry

Biocytin revelation was performed as previously described (Pannasch et al., 2011). Slices were fixed with 4% paraformaldehyde overnight, incubated in 1% Triton X-100 followed by revelation using Alexa Fluor 488-conjugated streptavidin (1/200 in PBS, Invitrogen). After several PBS washes, slices were mounted in Fluoromount (Southern Biotechnology) and examined with a confocal laser-scanning microscope (Leica TBCS SP2, SP5) with a 20× objective. Stacks of consecutive confocal images taken at 0.5 μm intervals were acquired with an argon laser (488 nm) and Z projections were reconstructed using Leica confocal Software. Cell counting was performed using ImageJ Software.

### Immunoblotting

For each condition, three hippocampal slices were frozen, pulverized, and homogenized in 2% SDS with protease inhibitor mixture, β-glycerophosphate (10 mM), and orthovanadate (1 mM). Equal amounts of protein were separated on a 10% PAGE gel followed by transfer to nitrocellulose membranes. Proteins were detected by immunoblotting using the HRP-ECL kit from Perkin-Elmer. GAPDH was used as loading control. Primary antibodies used were: vimentin rabbit polyclonal antibody (Chemicon), GFAP rabbit polyclonal antibody, Cx43 and Cx30 rabbit polyclonal antibodies (Zymed Laboratories). Donkey anti-rabbit IgG (Amersham Biosciences) HRP-conjugated secondary antibody was used.

### Three-Dimensional Confocal Morphometry

Fluorescently labeled astrocytes from GFAP-eGFP mice enable direct measurement of single astrocyte volume changes in acute brain slices using 3D-confocal morphometry, as previously described (Benesova et al., 2009, 2012; Anderova et al., 2014). Here we employed this technique for quantifying astrocyte volume changes *in situ*. Briefly, brain slices of GFAP-eGFP mice were placed in a chamber, perfused with ACSF at a rate of ~5 ml/min. Slices were kept in ACSF and modified ACSF solution was used to evoke astrocyte volume changes. All experiments were performed at room temperature (23–25°C). eGFP was excited with an Ar laser set at 488 nm, and the emitted signal was recorded over the range of 510–552 nm using a TD 488/543/633 filter. To minimize photo-bleaching, the laser intensity was always held at 25%. The signal was recorded using a Leica TCS SP system confocal microscope (Leica, Germany) with a water immersion 40× (0.8) HCX APO Leica objective (Leica, Germany). All data were acquired with Leica Confocal Software (Leica, Germany).

Astrocyte volume changes were determined from 3D images of individual astrocytes and every 3D image of the cell was sectioned into 70–80 consecutive two-dimensional (2D) images with a uniform spacing of 1 μm. The astrocytes were recorded as a set of 2D sectional images with an image size of 1024 × 1024 pixels. The scanning time for 70–80 2D images was ~150 s. The cell surface was found in each image using an edge-detecting algorithm, and the area of the image surrounded by the edge was calculated for each layer. The values of cell volume for individual cells were obtained by integrating the values of the edge length and area from all images in a set. Image processing and morphometric measurements were performed using the program CellAnalyst developed in the Department of Cellular Neurophysiology, IEM, Prague, Czech Republic.

### Calcium Imaging

Imaging experiments were performed simultaneously to electrophysiological field potential recordings in the same region of interest. Intracellular calcium measurements in astrocytes from hippocampal slices were made under single emission fluorescence microscopy using the fluorescent calcium indicator Fluo-4 AM (5 μM, Invitrogen), which has been demonstrated to load specifically astrocytes (Hirase et al., 2004). Loading was performed in ACSF for 45 min in the dark at 37°C. After recovery, slices were transferred to the recording chamber of an Olympus BX51WI microscope. Fluo-4 was excited at 488 nm through a light emitting diode (OptoLED, Cairn Research), controlled by the Axon Imaging Workbench software (Molecular Devices), triggering simultaneous acquisition of the electrophysiological recordings by Clampex10.3 software (Molecular Devices). Fluorescent light (>515 nm) emitted by labeled cells was detected with a long pass filter and an EM-CCD camera (Andor). Images were acquired at 1 Hz through a 20× water immersion objective (NA 0.95, Olympus) and stored on a PC. Images were processed and analyzed off-line with AIW imaging (Molecular Devices) and ImageJ Softwares. Background subtraction was performed prior to the fluorescent processing to correct for inhomogeneity of residual fluorescence due to the wide-field fluorescence microscope as well as the ongoing bleaching. Data were then expressed as relative changes in fluorescence over baseline (ΔF/F_0_). The degree of synchronization between fEPSP and astroglial calcium variations during exogenous glucose deprivation was quantified using cross-correlation analysis.

### Statistics

All data are expressed as mean ± SEM and *n* represents the number of independent experiments. Statistical significance was determined by one-way and two way repeated measures ANOVA followed by Bonferroni post-test, or *t*-test. Statistical analysis was performed using Statistica 6.1 and GraphPad Prism 6 Softwares.

## Results

### Glucose Contribution to Electrophysiological Properties of Astrocytes *In Situ*

**Figure 1:**
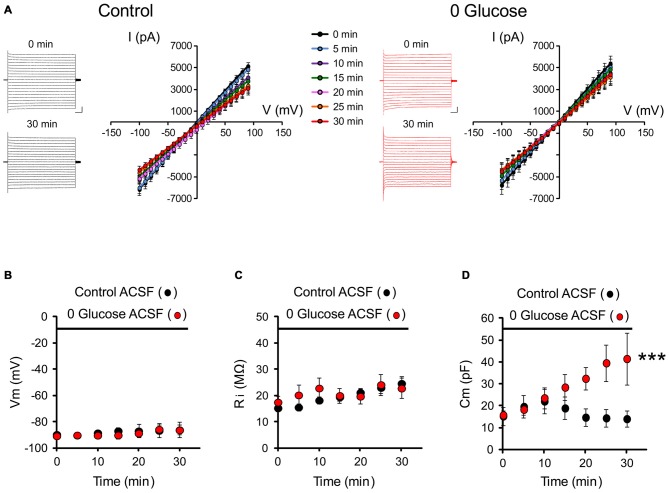
**Glucose deprivation alters astrocyte membrane capacitance. (A)** Representative traces of current-voltage relationships (IV curves) are illustrated before (0 min) and after 30 min of perfusion (30 min) with control (black traces) or 0 glucose artificial cerebrospinal fluid (ACSF; red traces). Scale bar, 2 nA, 50 ms. Mean current-voltage relationships (IV curves) recorded over 30 min are mostly unaltered by shortage of glucose (*n* = 8 cells, 8 slices, 4 mice) compared to control conditions (*n* = 7 cells, 7 slices, 5 mice, *p* > 0.05). **(B–D)** Both input resistance (Ri) and membrane potential (Vm) were indeed found to be unchanged (*n* = 8 cells, 8 slices, 4 mice) as compared to the control group perfused with glucose containing ACSF (*n* = 7 cells, 7 slices, 5 mice, *p* < 0.05). The only specific change in the astrocyte membrane properties during the course of glucose deprivation regards cell capacitance, which markedly increased in astrocytes exposed to glucose free (0 glucose, *n* = 8 cells, 8 slices, 4 mice) ACSF and not in control astrocytes exposed to regular glucose containing ACSF (*n* = 7 cells, 7 slices, 5 mice, *p* < 0.01). Asterisks indicate statistical significance (***p < 0.01).

To get insights into the consequences of energy deprivation on astroglial function, we first investigated whether glucose deprivation alters intrinsic electrophysiological properties of astrocytes. We recorded the typical passive membrane properties of astrocytes every 5 min for 30 min of glucose deprivation or regular ACSF (control) by analyzing the current or voltage responses to hyperpolarizing and depolarizing square pulses (I/V relationship illustrated in Figure 1A). We found no alteration in passive whole-cell current patterns (linear I/V relationship) during the course of glucose deprivation compared to the control group (control; *n* = 7; 0 glucose, *n* = 8; *p* > 0.05; Figure 1A). We also found no difference in the astrocyte resting membrane potential (Vm: control: −86.1 ± 6.0 mV, *n* = 7; 0 glucose: −86.3 ± 3.7 mV, *n* = 8 at 30 min; *p* > 0.05; Figure 1B) and input resistance (Ri: control: 24.6 ± 2.6 MΩ, *n* = 7; 0 glucose: 22.7 ± 3.9 MΩ, *n* = 8 at 30 min; *p* > 0.05; Figure 1C) compared to the control group. However, the capacitance specifically increased by more than 2.5 fold in astrocytes exposed to free glucose ACSF (Cm, at *t* = 0: 15.8 ± 2.0 nF; *t* = 30 min: 41.5 ± 11.8 nF, *n* = 8; *p* < 0.001), while it remained unchanged after 30 min in control cells (Cm, at *t* = 0: 15.2 ± 4.2 nF; *t* = 30 min: 14.0 ± 3.7 nF, *n* = 7; *p* > 0.05; Figure 1D). As the capacitance is classically considered to reflect cell volume, these data suggest that astroglial volume rapidly increases upon glucose deprivation.

### Glucose Deficiency Increases the Volume of Astrocytes

To directly examine the impact of acute glucose deprivation on the volume of astrocytes, brain slices from GFAP-eGFP mice were exposed to a 30 min application of glucose-free ACSF. Using 3D-confocal morphometry, we analyzed over the course of glucose deprivation the volume of GFP labeled astrocytes at the whole-cell and subcellular levels.

**Figure 2:**
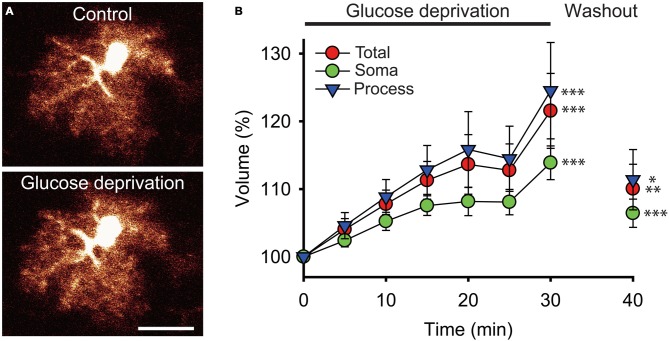
**Glucose deprivation increases astroglial volume. (A)** Sample confocal images of an enhanced green fluorescent protein (eGFP) labeled hippocampal astrocyte from a glial fibrillary acidic protein (GFAP)-eGFP mouse illustrated before (Control) and after 30 min of exogenous glucose deprivation. Scale bar, 10 μm. **(B)** Time-dependent changes in astrocytic total (red circles), soma (green circles) and processes (blue triangles) volumes were quantified using 3D confocal morphometry analysis in each individual cell every 5 min during exogenous glucose deprivation and after 10 min of washout. Volume changes were normalized to values measured at *t* = 0 and expressed relative to this baseline as an increase in percentage. Glucose deprivation increased significantly all astrocytic volumes, and this effect was partially reversible after 10 min of glucose re-introduction (*n* = 22 cells, 10 slices, 4 mice). Asterisks indicate statistical significance (*p < 0.05, **p < 0.01, ***p < 0.001).

We found that the total volume of astrocytes increased by ~22% during the 30 min glucose deprivation compared to the volume measured before glucose deficiency (peak increase at *t* = 30 min of glucose deprivation compared to *t* = 0: +21.5 ± 5.5%; *n* = 22; *p* < 0.001; Figures 2A,B). To determine whether the increase in astrocyte volume was homogenous throughout its domain, we measured the volume changes occurring locally in astrocyte soma and in processes that were induced by glucose deficiency. The volume of astrocyte somas, expressed as a fraction of the total cell volume, only increased by ~14% (+13.9 ± 2.5%, *n* = 22; *p* < 0.001), while volume changes in astrocytic processes were more pronounced, reaching ~25% after 30 min of glucose deficiency (+24.5 ± 7.1%; *n* = 22; *p* < 0.001; Figure 2B). Noteworthy, in response to glucose deprivation, the total volume of astrocytes displayed a pattern similar to the volume of their processes, consistent with the fact that the latter exceed by far the volume of the soma, and therefore accounts for most of astrocyte total volume. Over the course of glucose deprivation, the increases in astrocyte total and process volume were indeed consistently slightly stronger than the increase in soma volume, although the differences were not statistically significant (*p* > 0.05; Figure 2B).

We also found that the increase in astroglial compartments volume did not occur in a regular fashion during the course of glucose deprivation. Instead, all compartments initially displayed a constant and moderate rise during the first 15 min (processes and total volume: ~+4%/5 min; soma volume: ~+2.5%/5 min; *n* = 22), then stabilized over the next 10 min (increase at *t* = 25 min relative to increase at *t* = 15 min of glucose deprivation: processes and total volume: ~+1.5%; soma volume: ~+0.5%; *n* = 22), and finally showed a sharp increase during the last 5 min of glucose deprivation (processes and total volume: ~+10%/5 min; soma volume: ~+6%/5 min; *n* = 22; Figure 2B).

Noteworthy, the increases in astrocyte volume evoked by glucose deficiency were also all partially reversible. A 10 min washout performed after the 30 min of glucose deprivation by perfusing brain slices with regular glucose containing ACSF was indeed sufficient to induce a significant reduction in astrocyte total, soma and processes volume (total volume: ~−11.5%; process volume: ~−13%; soma volume: ~−7.5%; *n* = 22, *p* < 0.05; Figure 2B). Astrocytic volumes exposed to glucose-free ACSF nevertheless did not fully recover upon re-introduction of glucose, as they remained slightly higher than before deprivation (total volume: +10 ± 3.6%, *p* < 0.01; processes volume: +11.4 ± 4.5%, *p* < 0.05; soma volume: +5.4 ± 2.1%, *p* < 0.001; *n* = 22).

### Glucose Deficiency Impairs Gap-Junction Coupling of Astrocytes

**Figure 3:**
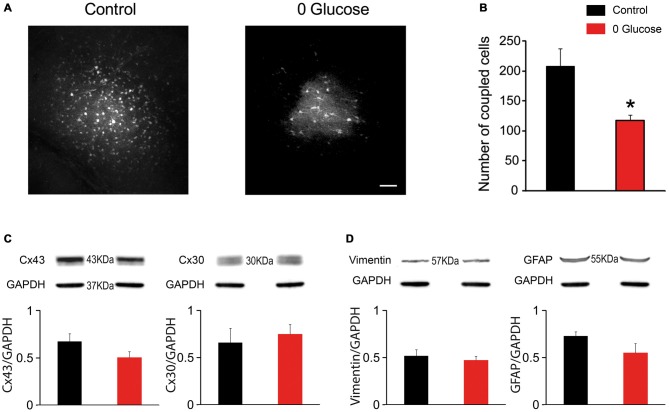
**Glucose deficiency impairs astrocyte gap junctional coupling. (A)** Sample images of gap junction-mediated biocytin coupling in CA1 *stratum radiatum* astrocytes from slices perfused with 30 min with control or 0 glucose ACSF. Scale bar, 50 μm. **(B)** Gap-junction coupling is significantly reduced following 30 min glucose deprivation, as quantified by counting the number of coupled cells following biocytin injection of a single astrocyte through a patch pipette in slices perfused with control (*n* = 5 slices, 3 mice) and 0 glucose ACSF (*n* = 5 slices, 3 mice, *p* < 0.05). **(C–D)** Western blot analysis of Cx43 and Cx30 **(C)**, GFAP and vimentin **(D)** in hippocampal slices exposed to control (*n* = 3 mice) and glucose-free ACSF for 30 min (*n* = 3 mice), showing no alteration in total protein levels. Asterisks indicate statistical significance (*p < 0.05).

One important property of astrocytes enabling them to spread metabolites across the neuropil is the high intercellular coupling they achieve through gap junctions. To assess whether shortage of the primary metabolite glucose affects the strong intercellular communication that takes place between hippocampal astrocytes, we infused biocytin, a low molecular weight tracer permeable to gap junction channels, specifically into a single astrocyte *via* a patch pipette during 30 min of glucose deprivation or regular ACSF (control). We observed an extensive intercellular diffusion of biocytin into the gap-junction mediated astroglial network, reaching more than 200 cells in control conditions (207.4 ± 30.0, *n* = 5). Remarkably, shortage of glucose during this relatively short period was sufficient to markedly reduce astroglial coupling, as the number of coupled cells significantly decreased by ~44% (117.4 ± 9.7, *n* = 5; *p* < 0.05; Figures 3A,B).

We then investigated whether the reduced astroglial coupling was mediated by a decrease in astroglial connexin expression. However, we found that acute glucose deprivation had no effect on total protein levels of both, Cx43 and Cx30 (*n* = 4, *p* > 0.05; Figure 3C). We also found that decreased coupling did not result from astroglial reactivity, since acute deprivation of glucose for 30 min did not alter GFAP and vimentin levels using western blot analysis (Figure 3D).

### Glucose Deprivation Simultaneously Alters Hippocampal Astroglial Calcium Levels and Excitatory Synaptic Transmission

**Figure 4:**
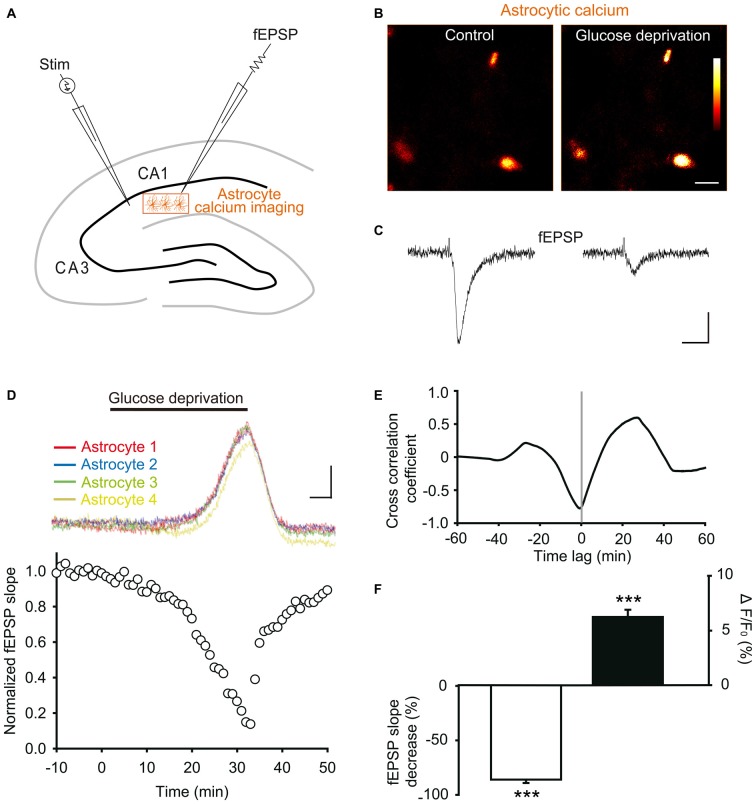
**Glucose deprivation increases intracellular calcium levels in astrocytes. (A)** Schematic depicting in a hippocampal slice dual recordings of field excitatory postsynaptic potentials (fEPSPs) evoked by Schaffer collaterals stimulation (Stim) and astroglial calcium levels. **(B–C)** Sample fluorescence images recorded in the orange zone shown in **(A)**, illustrating calcium levels of *stratum radiatum* astrocytes detected by Fluo-4 imaging **(B)**, and corresponding fEPSPs traces simultaneously recorded **(C)** before (Control) and after 30 min of glucose deprivation. Color bar: 0–255 (arbitrary fluorescence units, 8 bits resolution). Scale bars: 10 μm **(B)** and 0.2 mV, 20 ms **(C)**. **(D)** Quantification of simultaneous relative changes in astroglial calcium levels (ΔF/F_0_ in 4 color coded astrocytes, upper panel) and fEPSP slope (white circles, lower panel) induced by glucose deprivation in a representative experiment. Scale bar (upper panel), 2%, 5 min. **(E)** Cross correlation analysis between the time series of fEPSP and astroglial calcium signals illustrated in **(D)**. The high amplitude of the peak cross correlation coefficient (−0.78 at a time lag of −40 s) indicates a strong correlation between the two signals, where changes in fEPSP precede variations in astroglial calcium levels. **(F)** Quantification of mean peak relative changes in fEPSP slope and astroglial calcium levels (ΔF/F_0_) induced by the 30 min glucose deprivation (*n* = 5 cells, 5 slices, 3 mice). Asterisks indicate statistical significance (***p < 0.001).

A typical feature of astrocytes, besides passive membrane properties and extensive gap junction coupling, is their calcium signaling, thought to represent their excitability since these cells are electrically silent. Calcium signaling is indeed a characteristic response of astrocytes to local changes in synaptic activity, and can in turn regulate neurotransmission (Khakh and Mccarthy, 2015). We thus here investigated during the course of acute glucose deprivation the synchronous changes occurring in synaptic transmission of hippocampal CA1 pyramidal neurons and in intracellular calcium levels of adjacent astrocytes. To do so, we performed dual recordings of *stratum radiatum* astroglial calcium levels and fEPSPs evoked by Schaffer collateral stimulation (Figures 4A–C).

We found that exogenous glucose deprivation for 30 min induced a characteristic slow depression of excitatory synaptic transmission (Figure 4D), previously suggested to reflect exhaustion of glycogen stores (Wender et al., 2000). This depression was strongly correlated with an increase in astroglial calcium levels detected with the Fluo4 calcium indicator (Figures 4B–D), as indicated by the high amplitude of the cross correlation negative peak (correlation coefficient ~− 0.8, Figure 4E).

Peak fEPSP and calcium responses were both reached after ~30 min of glucose deprivation, and consisted in a marked depression (>80%) of synaptic transmission (normalized fEPSP slope: −86.0 ± 3.1%, *n* = 5, *p* < 0.001), and a moderate, but significant, increase in basal astrocyte calcium levels (ΔF/F0: +6.2 ± 0.7%, *n* = 5, *p* < 0.001, Figure 4F). The fEPSP peak decrease nevertheless preceded by ~40 s (time lag) the maximal astroglial calcium increase reached over the 30 min glucose deprivation, as revealed by the cross correlation analysis (Figure 4E). In addition, the slow kinetics of fEPSP depression was similar to the one of astroglial calcium increase (Figures 4D,E). Astroglial calcium and fEPSPs changes indeed only reached significance after ~20 min of glucose deprivation, time after which their variation sharply increased over the next 10 min (Figure 4D). Finally, both fEPSP and astroglial calcium alterations were reversible shortly after washout, recovering to almost baseline levels upon 10 min of exogenous glucose re-introduction (Figure 4D).

## Discussion

Brain adaptation to metabolic challenge is a fundamental process securing information processing and survival. Although glial cells play a prominent role in brain energy metabolism, how they cope with metabolic deficiency remains as yet poorly described. The present study therefore characterizes essential morphological and functional changes occurring in astroglial cells during shortage of the primary energy source in the central nervous system. Although no major alteration in the intrinsic whole-cell current pattern occurred, a marked augmentation in cell capacitance was found, suggesting an increase in astrocyte volume during glucose deprivation, a change that was confirmed by 3D-confocal morphometry analysis. Interestingly, this was accompanied by an impaired gap-junction mediated coupling of astroglial networks, as well as by an increase in intracellular calcium levels that correlated with an a strong reduction in excitatory synaptic transmission.

The explanation for the early glucose deprivation-induced increase in astrocyte volume likely lies in the marked reduction in gap junction coupling that we report. Indeed, poor astroglial coupling has previously been showed to increase cell volume in knock out animals for the gap-junction proteins connexins (Lutz et al., 2009; Pannasch et al., 2011; Chever et al., 2014). Double knock-out mice for both astroglial connexins Cx30 and Cx43, in which astroglial gap junction communication is abolished, show a large astroglial swelling (Lutz et al., 2009; Pannasch et al., 2011) due to intracellular accumulation of neurotransmitter and ions, mainly glutamate and potassium. The uncoupled cells indeed cannot distally redistribute elements accumulated during clearance of extracellular space following synaptic activity, thus leading to water co-entry to equilibrate intracellular osmolarity (Pannasch et al., 2011). Consistent with these data, we found that astrocytic processes, known to cover synaptic compartments (Hirrlinger et al., 2004), displayed a more pronounced swelling compared to soma. One possible explanation for these differential volume changes is the expression pattern of water channels aquaporin 4, well-known to be enriched in astroglial processes and endfeet (Nielsen et al., 1997; Smith and Verkman, 2015). Remarkably, relative compartmentalization of volume alterations has similarly been described in hippocampal neurons, for which cell body regions are more resistant to dynamic volume changes than adjacent dendritic regions (Andrew et al., 1997). The much less drastic impairment in astrocytic coupling we recently described in mice deleted for astrocytic Cx43 is more reminiscent of the reduction induced by glucose deprivation that we here report, and was found to increase astrocyte volume to a similar extent (~30%; Chever et al., 2014). The latter study also shows that the 50% reduction in coupling due to loss of Cx43 results in a ~100% increase in cell capacitance, a result clearly matching the astroglial profile we here observe during acute shortage of glucose. Most interestingly, in the latter study as in the data reported herein, the magnitude of volume vs. capacitance changes markedly differ. This may be due to the fact that the large change in capacitance (Cm) we report is likely contributed by a change in electrical accessibility of astroglial processes after swelling. Furthermore, capacitance is proportional to surface and not to volume, thus a 150% increase in the capacitance reflects a change in the surface but not in the volume (here 25%). Such strong surface increase may thus alternatively reflect expansion of very thin protrusions, which markedly augments cell surface, but contributes only little to cell volume.

Noteworthy, the change in astrocyte volume we report may be less pronounced *in situ* as the extracellular medium typically used in *ex vivo* preparation, as is the case herein, comprises higher glucose concentrations than reported *in vivo*. In addition, the volume change we report is unlikely to be the result of astroglial reactivity, since we found no alteration in GFAP and vimentin levels after 30 min of glucose deprivation, and the volume alterations were rapidly partially reversible upon glucose re-introduction (within 10 min). In addition, astroglial reactivity has been found to settle with a much longer time course (over months) in the context of glucose deregulation such as that occurring in diabetes (Lieth et al., 1998; Saravia et al., 2002). Furthermore we here showed that the volume of astroglial processes increase with glucose deprivation, whilst reactive astrocytes typically display a reduced ramification and therefore a decrease in overall process volume (Sun and Jakobs, 2012). Altogether, these elements suggest that the early morphological and functional changes occurring in astrocytes facing lack of glucose ensue from a reduced gap junction coupling. The latter reduction in coupling did not result from an alteration in connexin total protein levels. It however still remains that acute glucose deprivation may preferentially impair connexins engaged in functional gap junctions which contribute to only a minor part of the total connexin cellular pool. This may occur through rapid alteration of gap junctional conductances, post-translational modification of connexins, altered trafficking of connexins, disorganization of gap junctional plaques, or simply be the consequence of the change in mechanical stretch occurring as a result of an increase in cell volume as described in other systems (Salameh and Dhein, 2013). The hypothesis of changes in connexin levels at the membrane through alteration in trafficking in response to metabolic dysregulation is supported by a recent study showing impairment of astroglial connexins expression in experimental diabetes, a disease in which energy demand and supply frequently mismatch, thereby resulting in alternating episodes of hyper- and hypoglycemia (Gandhi et al., 2010; Ball et al., 2011). Interestingly in this study, Cx43 and Cx30 immunoreative punctate structures were reported to be selectively reduced by metabolic dysregulation, suggesting changes in trafficking of these connexins, which also induced a reduction of gap junction-mediated coupling. Alternatively, the decreased coupling induced by glucose deprivation may result from reduced conductance of astroglial connexins, as suggested by a recent study showing that Cx43 gap junction conductance is inhibited by ~50% in response to a short episode (5 min) of oxygen and glucose deprivation (Sahu et al., 2014).

Aside from the augmented cell capacitance, we found all intrinsic electrophysiological properties recorded to be unaltered, further demonstrating that gap junctional coupling does not account for the passive electrophysiological properties of astrocytes, notably their low membrane resistance (Schools et al., 2006; Pannasch et al., 2011; Chever et al., 2014), originally thought to be attributable to the formation of a syncytium between astrocytes. Several lines of evidence have indeed now shown that astrocyte passive conductances are intrinsic properties of their membrane expressing specific ions channels, notably the K2P potassium channel heterodimers TWIK-1/TREK-1 (Schools et al., 2006; Zhou et al., 2009; Hwang et al., 2014; Du et al., 2015).

Although the latter intrinsic passive properties of astrocytes are mostly unaffected by glucose deprivation, a sudden reduction in vital nutrients is known to result in ionic imbalance and changes in neurotransmitters concentrations, such as that occurring during ischemic episodes, including increase in extracellular glutamate and potassium, concomitant to a raise of intracellular calcium and sodium (Lipton, 1999). Cell culture data have showed that the sole removal of glucose from the extracellular medium increases intracellular calcium and sodium, whilst it decreases potassium both in neurons and astrocytes (Silver et al., 1997; Arnold, 2005). Although the opposite changes in sodium vs. potassium concentrations occur within only 10 min, it is likely to reflect the failure of both cell types to maintain proper ionic balance due to energy shortage. Our finding that membrane potential and passive physiological properties of astrocytes from hippocampal slices remained unchanged during glucoprivation is therefore puzzling. This may however be explained by the fact that glucoprivation leads to rapid astrocytes ATP depletion, intracellular acidification (ATP hydrolysis releases a H^+^) as well as Na^+^ accumulation (Fernández-Moncada and Barros, 2014). Acidification will indeed eventually lead to hyperpolarization through activation of sodium-bicarbonate co-transporter (NBC) and sodium-hydrogene exchanger (NHE) as astrocytic NBC co-transports 2 HCO_3_^−^ for every Na^+^. This might help to mask the slight depolarization induced by loss of intracellular K^+^. In a future investigation, it would thus be interesting to monitor changes in intracellular [Na^+^], [ATP] and pH during glucoprivation. The increase in intracellular calcium however remains and correlates with the marked reduction in synaptic efficacy.

Whether enhanced calcium levels are a consequence and/or a cause of synaptic transmission impairment still remains to be determined. Increased astroglial calcium levels may simply result from ionic imbalance associated with metabolic deficiency, or involves specific active mechanisms. One possibility is that exogenous glucose deprivation, by removing fuel for glycolysis, may dramatically decrease ATP production, thereby inhibiting calcium ATPase pumps in the endoplasmic reticulum, resulting in calcium release from internal stores. This process may be accelerated by intracellular glucose extrusion *via* GLUT2 glucose transporters when extracellular levels fall below intracellular concentrations, as recently hypothesized (McDougal et al., 2013). Another intriguing possibility may involve direct signaling mediated by low glucose sensing via GLUT2 transporters, which also act as receptors capable of regulating food intake (Stolarczyk et al., 2010). These hypotheses are however to be mitigated by the fact that astroglial expression of GLUT2 is not prominent in the hippocampus (Arluison et al., 2004).

Interestingly, elevation of astrocytic calcium levels during glucoprivation may tune synaptic transmission by various mechanisms. It may serve glycogenolysis, as shown in human astrocytoma cells (Medrano et al., 1992), to sustain synaptic activity by local supply of glucose to neurons. It might also represent an active process enabling to directly tune down synaptic efficacy to preserve energy stores by promoting gliotransmitter release such as ATP. ATP is indeed well known to be rapidly metabolized extracellularly into adenosine, which, through activation of presynaptic A1 receptors, inhibits presynaptic glutamate release (Pascual et al., 2005). Future investigation analyzing calcium variation in distal astrocyte processes using mice expressing the ultrasensitive protein calcium sensors *GCaMP6* may provide further insights on these processes.

Interestingly, glucoprivation induced changes in astrocytic calcium has recently been reported in astrocytes from the nucleus of the solitary tract (NST) in the dorsal medulla, a brain region that has long been associated with central detection of glucose availability and control of glucose homeostasis (McDougal et al., 2013). Insofar as NST astroglia also responded to low glucose (2.5, 1, and 0.5 mM) and retrieved baseline calcium concentration upon return to normal glucose level, authors proposed that these astrocytes may represent a new type of glucosensor, in addition to the well described ventromedian hypothalamic neurons that are either excited (GE neurons) or inhibited (GI neurons) by glucose (Penicaud et al., 2006). In accordance with such conclusion, a subsequent study found that inhibiting NST astrocytes with fluorocitrate impairs the gastric vagal reflex circuits and gastric motility induced by glucoprivation (Hermann et al., 2014). Our study supports and extends this view by showing that astrocytes from a brain region that is not classically associated with glucose homeostasis also respond to a change in glucose availability. It is therefore conceivable that glucosensing is a widespread competence of astrocytes, which in response to hypo- or hyper-glycemia could orchestrate local management of limited or excessive energy resources. In that respect, the markedly reduced astroglial coupling we report herein could represent a mean of limiting diffusion of the poor store of energy molecules in order to promote survival of the local network.

In conclusion, the present dataset suggests that astrocytes throughout the brain play a pivotal role in managing brain adaptation to glucose deficiency. Although caution should be used when interpreting acute vs. chronic metabolic challenge and tissue from different ages, our findings are nonetheless relevant to our understanding of the processes taking place in the ageing nervous system, frequently challenged with glycopenia. Further implication of the present work include pathological conditions associated with glucose deficiency, such as glucose transporter-1 deficiency syndrome in which the function of the main blood brain barrier glucose transporter GLUT1 is lost (Wang et al., 2005), as well as Alzheimer’s disease in which GLUT1 downregulation plays a significant role (Winkler et al., 2015).

## Author Contributions

C-YL, GD, MA and NR: conception and experimental design, methodology and data acquisition, analysis and interpretation of data. GD and NR: manuscript writing.

## Conflict of Interest Statement

The authors declare that the research was conducted in the absence of any commercial or financial relationships that could be construed as a potential conflict of interest.
